# Analysis of latent profiles and influencing factors of information overload in day surgery patients

**DOI:** 10.3389/fpubh.2026.1844987

**Published:** 2026-08-04

**Authors:** Liyan Qiu, Mei Yang, Feng Su, Liping Ma, Yanying Wang, Bixue Chen, Zuqiong Ye, Longyan Wu

**Affiliations:** Department of Day Surgery Ward, The First Affiliated Hospital of Guangxi Medical University, Nanning, Guangxi, China

**Keywords:** cross-sectional study, day surgery, influencing factors, information overload, latent profile

## Abstract

**Background:**

Day surgery has become an increasingly important model of healthcare delivery and offers clear benefits to patients. However, because hospitalization, surgery, and discharge occur within a very short period, patients are required to absorb a large amount of medical information in a limited timeframe, making them particularly susceptible to information overload. Although this phenomenon has received growing attention, little remains known about the latent subgroups of information overload among patients undergoing day surgery. This study therefore aimed to identify latent profiles of information overload and examine their associated factors using latent profile analysis (LPA).

**Methods:**

A cross-sectional survey was performed between October and December 2024 among 347 hospitalized day surgery patients in China. Participants completed the Chinese versions of the Information Overload Scale, Information Self-Efficacy Scale, and Electronic Health Literacy Scale. LPA was used to classify patients according to their information overload patterns, and multinomial logistic regression was used to identify factors related to membership in different latent profiles.

**Results:**

Overall, patients experienced a moderate level of information overload. LPA identified three distinct subgroups: a low information overload group (*n* = 193, 55.6%), a moderate information overload group (*n* = 113, 32.6%), and a high information overload group (*n* = 41, 11.8%). Multivariable logistic regression demonstrated that age, education level, occupational category, and surgical specialty were independent predictors of latent profile membership (*p* < 0.05).

**Conclusion:**

Heterogeneity in information overload was noted among day surgery patients, and was categorized into three distinct latent profiles. Identifying patients at greater risk and implementing targeted interventions to reduce information burden may improve information management abilities and support enhanced postoperative recovery.

## Introduction

Day surgery has been established as an increasingly adopted healthcare model worldwide. Under this approach, patients complete admission, surgery, and discharge within a 24-h period (1). In China, the expansion of day surgery has become an important component of public hospital reform, with the goals of improving healthcare efficiency, optimizing resource utilization, and enhancing patient experience (2). Despite these advantages, the condensed nature of day surgery requires patients to receive extensive preoperative, intraoperative, and postoperative information over a very short period. When the volume and complexity of information exceeds effective individual processing capacity, information overload may occur, resulting in cognitive burden and impaired information processing (3, 4).

Information overload is common among patients with various medical conditions and is associated with adverse clinical and psychological outcomes. Among individuals with cancer, excessive health information has been linked to difficulties in managing information, increased anxiety and uncertainty, and reduced willingness to seek additional health information (5, 6). Similar findings have been reported in patients with chronic diseases, in whom information overload has been associated with poorer treatment adherence and reduced self-management capacity (7, 8). However, previous research has focused primarily on patients receiving long-term care, such as those with cancer or chronic illnesses, whereas information overload in the context of day surgery has received relatively little attention.

Information overload is particularly relevant in the day surgery setting because of several unique characteristics. First, patients must receive comprehensive information regarding preoperative preparation, surgical procedures, postoperative care, medication management, rehabilitation, and discharge instructions within a highly compressed timeframe. This intensive educational process differs substantially from the gradual information delivery typically provided during conventional inpatient hospitalization. Second, because patients return home shortly after surgery, they must independently manage postoperative recovery without continuous professional supervision. Inadequate comprehension or retention of essential discharge information may negatively affect home-based recovery, delay recognition of postoperative complications, and ultimately compromise clinical outcomes (9, 10). Consequently, understanding the extent and determinants of information overload in this population is essential for developing more effective perioperative education strategies and promoting enhanced recovery after surgery.

Latent profile analysis (LPA) is a person-centered statistical technique that identifies unobserved population subgroups based on patterns of responses across multiple observed variables, with the optimal number of profiles determined using model fit statistics (11, 12). Unlike conventional variable-centered analytical methods, LPA captures population heterogeneity by classifying individuals with similar response patterns into distinct latent profiles. This can help reveal differences in information overload and facilitates the development of targeted intervention strategies for specific patient subgroups. To our knowledge, no previous study has applied LPA to characterize information overload among patients undergoing day surgery.

Accordingly, this study employed LPA to identify distinct patterns of information overload among day surgery patients and to determine the factors associated with membership in each profile. These results will provide an evidence-based foundation for individualized information support strategies, thereby improving perioperative patient education and facilitating enhanced postoperative recovery.

## Materials and methods

### Study design

A cross-sectional study was conducted. Sample size estimation followed the recommendation for descriptive cross-sectional studies involving quantitative variables (13), whereby the required sample size should be approximately 5–10 times the number of independent variables included in the analysis. Because 13 independent variables were considered, the minimum required sample size was estimated to be 65–130 participants. To compensate for an anticipated invalid response rate of approximately 20%, the target sample size was increased, and a total of 347 participants were ultimately included in the study.

### Participants

The Medical Ethics Committee of the First Affiliated Hospital of Guangxi Medical University gave study approval. Participants were recruited using convenience sampling between October and December 2024. All study subjects were recruited from the same day surgery ward, rather than a conventional inpatient surgical ward. Day surgery patients underwent a relatively standardized management protocol, including preoperative evaluation, perioperative care, short-term postoperative observation, discharge assessment, and post-discharge follow-up. The inclusion criteria were: (a) patients meeting the eligibility criteria for day surgery; (b) patients capable of completing the questionnaire either independently or with assistance; (c) patients and their families providing written informed consent and voluntarily participating in the survey. The exclusion criterion was: patients who withdrew from day surgery or were transferred to specialized wards for treatment during the perioperative period.

Ultimately, 347 eligible patients were enrolled in the study.

## Questionnaire survey

### General characteristics

Demographic, socioeconomic, and clinical information was collected, including age, gender, marital status, education level, occupation, per capita monthly family income, medical payment method, presence and number of comorbidities, and surgical department.

### Information Overload Scale

Information overload was assessed using the Chinese version of the Information Overload Scale. The original instrument was developed by Jensen et al. (14) and subsequently translated, culturally adapted, and validated for Chinese populations by Ding Yaoyao et al. (15). The scale evaluates patients’ perceived information overload during hospitalization. The instrument comprises eight items forming a single-dimensional construct. Each item is rated on a four-point Likert scale from 1 (“strongly disagree”) to 4 (“strongly agree”), for a total score of 8 to 32. Higher scores are indicative of greater perceived information overload. Total scores of 8–14 represent low information overload, scores of 15–22 indicate moderate information overload, and scores of 23–32 reflect high information overload. The original Chinese version presented with a high degree of internal consistency, with a Cronbach’s alpha coefficient of 0.884, while the reliability observed in the present study was even higher, with a Cronbach’s alpha of 0.956.

### Information Self-Efficacy Scale

Information self-efficacy was assessed with the Information Self-Efficacy Scale developed by Zhang Wei et al. (16). The instrument measures patients’ confidence in their ability to obtain, understand, and manage health-related information. It consists of three items rated on a five-point Likert scale, with response options ranging from 1 (“strongly disagree”) to 5 (“strongly agree”). Higher total scores indicate greater perceived self-efficacy in managing health information. The reported Cronbach’s alpha coefficient for the scale is 0.751.

### eHealth Literacy Scale (eHEALS)

Electronic health literacy was measured using the Chinese version of the eHEALS, which was culturally adapted and validated by Guo Shuaijun et al. (17). The scale comprises eight items across three domains: the ability to locate and use online health information, evaluate the quality of online health resources, and make informed health decisions based on electronic information and services. Each item is rated on a five-point Likert scale ranging from 1 (“strongly disagree”) to 5 (“strongly agree”). Total scores range from 8 to 40, with higher scores reflecting greater electronic health literacy. The scale has demonstrated excellent internal consistency, with a Cronbach’s alpha coefficient of 0.913.

### Data collection and quality control

Before recruitment, all members of the research team completed standardized training covering the study objectives, survey procedures, interpretation of questionnaire items, standardized administration techniques, and patient communication skills. Only investigators who successfully completed the training assessment were authorized to conduct data collection. Questionnaire administration began after patients were admitted for day surgery. Using a standardized script, investigators explained the study purpose, emphasized the voluntary nature of participation, assured participants of data confidentiality, and obtained written informed consent from patients and their families. Whenever possible, questionnaires were completed independently by participants. For patients who were unable to complete the survey without assistance, trained investigators read each question verbatim in a neutral, non-leading manner and recorded responses only after obtaining the participant’s permission. All questionnaires were collected on the day of discharge. A designated research assistant immediately reviewed each questionnaire for completeness. If any responses were missing, participants were asked to complete the omitted items before leaving the hospital whenever feasible. Questionnaires containing more than 10% missing data were considered invalid and excluded from the final analysis.

To ensure data quality, two independent researchers entered all questionnaire data separately. The two datasets were subsequently cross-checked, and logical consistency checks were performed to identify and correct any discrepancies. Throughout the data collection period, the research team held regular meetings to discuss operational issues encountered during fieldwork and to standardize survey procedures, thereby minimizing measurement error and information bias.

### Statistical analysis

Latent profile analysis was conducted in Mplus v8.3 to identify underlying subgroups of information overload among patients undergoing day surgery. Individual scores for the eight items of the Information Overload Scale served as indicator variables in the LPA models. Models containing one to five latent profiles were estimated sequentially, and the optimal solution was selected based on overall model fit and statistical criteria. Model fit was evaluated using the Akaike Information Criterion (AIC), Bayesian Information Criterion (BIC), sample-size adjusted BIC (aBIC), entropy, Lo–Mendell–Rubin likelihood ratio test (LMR), and bootstrap likelihood ratio test (BLRT). Better model fit is indicated by lower AIC, BIC, and aBIC values. Entropy ranges from 0 to 1, with better classification accuracy corresponding to higher values. Significant LMR and BLRT results (*p* < 0.05) indicate that a model with *k* latent classes provides a significantly better fit than a model with *k* − 1 classes. Subsequent statistical analyses were performed using IBM SPSS Statistics 26.0 (IBM Corp., Armonk, NY, USA). Demographic characteristics, socioeconomic variables, and disease-related information were summarized using descriptive statistics, including means, standard deviations, frequencies, and percentages. Differences in categorical variables among latent profile groups were evaluated using the chi-square (χ^2^) test. Variables associated with latent profile membership were further examined using multinomial logistic regression analysis. All statistical tests were two-sided, and *p* < 0.05 was considered significant.

## Results

### Participant characteristics

A total of 350 questionnaires were distributed and returned during the study period. After excluding three questionnaires that did not meet the quality criteria, 347 valid questionnaires remained for analysis, yielding a 99.14% effective response rate. Among the participants, 63.9% were between 18 and 44 years of age, 70.9% were married, and 54.5% had attained a college education or higher. The enrolled patients underwent various types of day surgery, with the proportions as follows: Patients from the orthopedic surgery patients for 141/347 (40.6%) gastrointestinal glandular surgery department accounted for 113/347 (32.6%), urology surgery patients for 54/347 (15.6%), and hepatobiliary surgery patients for 39/347 (11.2%), among which orthopedic surgery accounted for the highest proportion. Despite variations in patient conditions and surgical types, all patients received standardized perioperative and post-discharge management protocols within the same day surgery unit. Additional demographic and clinical characteristics are summarized in Table 1.

**Table 1:** Single factor analysis of three potential profiles of information overload.

Variables	C1 (*n* = 193) *n* (%)	C2 (*n* = 113) *n* (%)	C3 (*n* = 41) *n* (%)	χ^2^	*P*
Gender				1.213	0.545
Male	107 (55.4)	58 (51.3)	25 (61.0)		
Female	86 (44.6)	55 (48.7)	16 (39.0)		
Age (year)				59.975	<0.001
18–44	151 (78.2)	58 (51.3)	13 (31.7)		
45–59	40 (20.7)	48 (42.5)	16 (39.0)		
≥60	2 (1.0)	7 (6.2)	12 (29.3)		
Marital status				22.572	<0.001
Unmarried	67 (34.7)	21 (18.6)	2 (4.9)		
Married	120 (62.2)	88 (77.9)	38 (92.7)		
Widowed or separated	6 (3.1)	4 (3.5)	1 (2.4)		
Education level				189.183	<0.001
Primary school and below	2 (1.0)	18 (15.9)	16 (39.0)		
Junior high school	6 (3.1)	50 (44.2)	15 (36.6)		
High school	10 (5.2)	32 (28.3)	9 (22.0)		
College degree or above	175 (90.7)	13 (11.5)	1 (2.4)		
Occupation				101.920	<0.001
Staff	111 (57.5)	17 (15.0)	2 (4.9)		
Self-employed individual	10 (5.2)	16 (14.2)	4 (9.8)		
Farmers	17 (8.8)	46 (40.7)	23 (56.1)		
Others	55 (28.5)	34 (30.1)	12 (29.3)		
Per capita monthly family income				26.576	<0.001
<3000 yuan	50 (25.9)	54 (47.8)	22 (53.7)		
3,000–5000 yuan	85 (44.0)	40 (35.4)	17 (41.5)		
>5000 yuan	58 (30.1)	19 (16.8)	2 (4.8)		
Medical payment method				57.718	<0.001
Urban and rural residents	49 (25.4)	68 (60.2)	27 (65.9)		
Employee medical insurance	132 (68.4)	34 (30.1)	9 (22.0)		
Self-funded	12 (6.2)	11 (9.7)	5 (12.2)		
Place of residence				57.541	<0.001
Rural area	44 (22.8)	70 (61.9)	27 (65.9)		
City	149 (77.2)	43 (38.1)	14 (34.1)		
Comorbidities				21.650	<0.001
None	182 (94.3)	93 (82.3)	29 (70.7)		
Have	11 (5.7)	20 (17.7)	12 (29.3)		
Number of comorbidities				24.866	<0.001
0	181 (93.8)	91 (80.5)	28 (68.3)		
1 type	11 (5.7)	17 (15.0)	11 (26.8)		
2 ~ 3 types	1 (0.5)	5 (4.4)	2 (4.9)		
Surgical department				52.587	<0.001
Gastrointestinal glandular surgery	73 (37.8)	36 (31.9)	4 (9.8)		
Orthopedics	91 (47.2)	39 (34.5)	11 (26.8)		
Urology surgery	23 (11.9)	17 (15.0)	14 (34.1)		
Hepatobiliary surgery	6 (3.1)	21 (18.6)	12 (29.3)		
Information self-efficacy	11.83 ± 1.83	8.96 ± 2.21	8.27 ± 2.46	97.728	<0.001
eHEALS	31.35 ± 5.32	24.20 ± 5.64	22.20 ± 6.75	82.231	<0.001

### Latent profiles of information overload

An optimal latent profile solution was determined by estimating models containing one to five latent classes, with corresponding goodness-of-fit statistics being presented in Table 2. The LMR test indicated that the 2-, 3-, and 4-profile models (all LMR *p* < 0.01) but non-significant for the 5-profile model (LMR *p* = 0.257). Although the four-profile solution demonstrated slightly lower AIC, BIC, and aBIC values and marginally higher entropy than the three-profile model, the improvement in overall fit was limited. Moreover, the smallest subgroup in the four-profile solution accounted for only 11.32% of the sample, approaching the lower limit generally considered desirable for stable profile estimation. The five-profile model performed less favorably, containing a very small subgroup (7.70%) and failing the LMR test. Considering statistical fit, classification accuracy, subgroup stability, interpretability, and clinical applicability, the three-profile model was chosen as the final solution.

**Table 2:** Fit statistics of the latent profile analysis (*n* = 347).

Model	AIC	BIC	aBIC	LMR	BLRT	Entropy	Proportion
1-profile	7374.009	7435.598	7384.841	–	–	–	–
2-profile	5712.667	5808.900	5729.593	<0.001	<0.001	0.954	0.66115/0.33885
3-profile	5143.352	5274.229	5166.370	<0.001	<0.001	0.963	0.55620/0.32565/0.11816
4-profile	4567.490	4733.011	4596.602	0.0056	<0.001	0.973	0.24578/0.40091/0.24015/0.11316
5-profile	4424.174	4624.339	4459.379	0.2571	<0.001	0.976	0.19056/0.38535/0.07698/0.23401/0.11310

**Figure 1:**
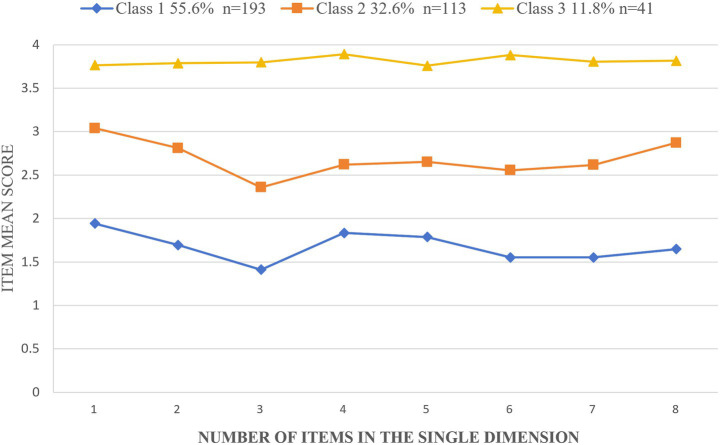
Potential profile of information overload. C1: Low information overload group, C2: Moderate information overload group, C3: High information overload group.

As shown in Figure 1, the three latent profiles demonstrated distinct patterns across the eight Information Overload Scale items. The mean total information overload scores for Classes 1, 2, and 3 were 13.42 (SD = 2.87), 21.53 (SD = 2.23), and 30.44 (SD = 2.05), respectively. Based on the established cutoff values of the Chinese version of the Information Overload Scale (8–14 = low, 15–22 = moderate, and 23–32 = high), Class 1 was designated the Low Information Overload group, with 193 participants (55.6%); Class 2 was designated the Moderate Information Overload group, with 113 participants (32.6%); and Class 3 was designated the High Information Overload group, with 41 participants (11.8%).

### Scores and correlations among information overload, information self-efficacy, and eHealth literacy

Among the 347 participants, the mean information overload score was 18.07 (SD = 6.39). The mean information self-efficacy score was 10.48 (SD = 2.54), while the mean eHealth literacy score was 27.94 (SD = 6.79). Correlation analysis demonstrated that information overload was significantly and negatively associated with both information self-efficacy (*r* = −0.605, *p* < 0.001) and eHealth literacy (*r* = −0.564, *p* < 0.001), indicating that patients with greater confidence in managing health information and higher levels of electronic health literacy were less likely to experience information overload.

### Univariate analysis of the three latent profiles of information overload

The univariate comparisons among the three latent information overload profiles are presented in Table 1. Significant differences were observed across the three groups with respect to age, marital status, education level, occupation, per capita monthly family income, medical payment, place of residence, presence of comorbidities, number of comorbidities, surgical department, information self-efficacy, and eHealth literacy (all *p* < 0.05).

### Multivariate analysis of factors related to latent profile membership

For factors significant in univariate analyses (*p* < 0.05), their significance in a multinomial logistic regression model was examined to identify independent predictors of latent profile membership. The high-information-overload group served as the reference category, and profile membership was treated as the dependent variable. Predictor variables included age, marital status, education level, occupation, per capita monthly family income, medical payment, place of residence, presence of comorbidities, number of comorbidities, surgical department, information self-efficacy, and eHealth literacy. The regression results are summarized in Table 3, with significant predictors highlighted in bold. Age, education level, occupation, and surgical department were identified as independent factors associated with latent profile membership. Specifically, patients aged 45–59 years were more likely to belong to the moderate-information-overload group, whereas those with lower education level had a greater likelihood of being classified in the high-information-overload group. In contrast, office employees and patients undergoing gastrointestinal or urologic surgery were more likely to be assigned to the low-information-overload group.

**Table 3:** Multinomial logistic regression analysis of the latent profile membership (n = 347).

**Variables**	C1: Low information overload group (vs. C3: High information overload group)			
	*B*	SE	*P*	OR(95%CI)
Education level (ref: College degree or above)				
Primary school and below	−7.943	1.970	<0.001	0.0004 (0.0000075,0.0168)
Junior high school	−6.424	1.539	<0.001	0.002 (0.0000795,0.0332)
High school	−5.562	1.428	<0.001	0.004 (0.000233,0.0630)
Occupation (ref: Others)				
Staff	2.561	1.296	0.048	12.947 (1.02,164.2)
Self-employed individual	0.252	1.190	0.832	1.286 (0.125,13.25)
Farmers	0.514	0.831	0.536	1.672 (0.328,8.53)
Surgical department (ref: Hepatobiliary Surgery)				
Gastrointestinal glandular surgery	3.618	1.354	0.008	37.249 (2.62,529.0)
Orthopedics	1.786	1.308	0.172	5.964 (0.459,77.4)
Urology Surgery	2.761	1.346	0.040	15.821 (1.13,221.2)

**Variables**	C2: Moderate information overload group (vs. C3: High information overload group)			
	*B*	SE	*P*	OR(95%CI)
Age (year) (ref: ≥60)				
18–44	1.536	0.844	0.069	4.646 (0.889, 24.296)
45–59	1.981	0.741	0.007	7.248 (1.697, 30.971)
Education level (ref: College degree or above)				
Primary school and below	−3.387	1.589	0.033	0.034 (0.001, 0.761)
Junior high school	--1.840	1.372	0.180	0.159 (0.011, 2.341)
High school	−1.241	1.335	0.353	0.289 (0.021, 3.958)
Surgical department (ref: Hepatobiliary Surgery)				
Gastrointestinal glandular surgery	1.613	0.792	0.042	5.019 (1.063, 23.685)
Orthopedics	1.215	0.899	0.177	3.370 (0.578, 19.665)
Urology Surgery	0.184	0.606	0.761	1.202 (0.366, 3.949)

## Discussion

The mean information overload score among the 347 day surgery patients in this study was 18.07 ± 6.39, indicating an overall moderate level of information overload. This value was lower than those previously reported in patients with atrial fibrillation (15) and cancer (18). The difference is likely attributable to variations in patient characteristics. Individuals with chronic diseases or malignancies must cope with multiple treatment options, prolonged rehabilitation, and uncertainty regarding prognosis, all of which substantially increase the volume and complexity of health information they are required to process (5, 6, 11, 12). In contrast, candidates for day surgery are typically selected according to strict preoperative criteria, including an ASA physical status of I or II and well-controlled comorbidities, resulting in relatively uncomplicated clinical conditions, standardized treatment pathways, and more focused informational needs, thereby reducing the likelihood of information overload (19). Furthermore, the implementation of intelligent, multimodal perioperative education platforms in many healthcare institutions may have further improved the efficiency of information delivery (20). Nevertheless, although the overall level of information overload appeared moderate, LPA demonstrated considerable heterogeneity within the study population. Specifically, 11.8% of patients were classified into the high-information-overload subgroup, while an additional 32.6% belonged to the moderate-overload subgroup, indicating that nearly one-half of all day surgery patients experienced clinically meaningful information burden. Older adults and individuals with lower education level appeared to derive less benefit from existing educational approaches (21). Consequently, relying solely on the overall mean score may obscure important subgroup differences and underestimate the needs of vulnerable patients. This finding highlights the value of LPA as a means of uncovering clinically relevant heterogeneity that cannot be identified using conventional summary statistics.

Latent profile analysis identified three distinct patterns of information overload among day surgery patients: low, moderate, and high overload. These subgroups demonstrated clear gradients in demographic and cognitive characteristics. The low-overload group consisted primarily of younger adults (18–44 years) with at least a college education, who generally exhibited stronger health literacy and greater capacity to obtain, interpret, and apply medical information (22). The moderate-overload group was composed predominantly of middle-aged individuals (45–59 years) with junior high school or high school education. Although these patients possessed a basic ability to process health information, relatively limited learning capacity and cognitive resources made it more challenging to assimilate large amounts of unfamiliar information, resulting in a moderate degree of overload (23). In contrast, the high-overload group was largely comprised of patients aged 60 years or older with an elementary school education or less. Age-related cognitive decline combined with limited educational attainment likely reduced their ability to understand and retain health information, predisposing them to severe information overload (24). These findings are consistent with the latent profile classifications reported by Zhang et al. (18) in patients with cancer, suggesting that information overload may exhibit comparable latent structures across diverse clinical populations and providing a useful framework for future comparative research.

Unlike traditional variable-centered statistical approaches, which primarily identify factors associated with an outcome, LPA classifies individuals into clinically meaningful subgroups based on shared response patterns. This person-centered approach therefore provides information that is directly applicable to individualized clinical management (25, 26). To our knowledge, this is the first study to characterize latent patterns of information overload specifically among day surgery patients. More importantly, it identified a readily recognizable high-risk subgroup, characterized by advanced age and lower education level, thereby providing clinicians with practical criteria for identifying patients most likely to experience excessive information burden. These findings support a transition from standardized health education toward risk-stratified, individualized information support tailored to the specific needs of different patient groups, representing an important clinical contribution of the present study.

Based on the distinct characteristics of the three latent profiles, we propose a tiered approach to perioperative information delivery. For patients in the low-overload group, who generally possess strong information-processing abilities, educational efforts should emphasize efficiency rather than quantity. A concise education strategy focusing on essential topics, including the surgical timeline, discharge criteria, and emergency contact information, may be sufficient, while avoiding unnecessary details that could impose additional cognitive burden. These patients can also be encouraged to obtain supplementary educational materials independently through digital platforms and to participate actively in shared decision-making (27, 28). Patients in the moderate-overload group would likely benefit from a structured education strategy. Medical information should be presented sequentially according to the perioperative timeline, including preoperative preparation, intraoperative procedures, and postoperative recovery. Complex concepts should be simplified through the use of illustrations, flowcharts, and short educational videos to facilitate comprehension and reduce cognitive load. In addition, nurses should reinforce key messages and confirm patient understanding at critical perioperative time points to improve communication and information retention (29–31). For patients in the high-overload group, whose cognitive processing capacity and learning ability are substantially more limited, educational interventions should prioritize simplicity, repetition, and caregiver involvement. Family members should be actively included in health education sessions, with face-to-face explanations supplemented by concise written instructions. At least two structured review sessions should be completed before discharge to verify understanding of essential postoperative instructions. Following discharge, gradual reinforcement of rehabilitation information through scheduled telephone or WeChat follow-up on postoperative days 1 and 7 may improve adherence while avoiding excessive information delivery during a single encounter. When appropriate, coordination with community healthcare providers may further facilitate home-based rehabilitation and continuity of postoperative care (32–35).

The present study further demonstrated that both age and educational attainment are important determinants of information overload among patients undergoing day surgery, consistent with the findings reported by Zhang et al. (18). Several mechanisms may account for these associations. First, patients with lower educational attainment generally have less efficient cognitive processing, including reduced reading comprehension and analytical skills (36). Consequently, they may have difficulty interpreting medical terminology and integrating fragmented perioperative instructions within the compressed timeline of day surgery. Second, these individuals often exhibit deficits in health literacy at multiple levels. In addition to limited foundational medical knowledge, they may have reduced ability to evaluate and filter health information effectively, thereby diminishing the quality of information available to support clinical decision-making (37). Third, advancing age is frequently accompanied by declining cognitive function and inadequate digital health literacy. The combination of these limitations creates a shortage of both cognitive and technological resources, increasing susceptibility to information overload (38). Occupational status was also identified as an independent influencing factor. Compared with other occupational groups, office employees were more likely to belong to the low-information-overload profile, possibly because they generally possess higher educational attainment, stronger information literacy, and broader opportunities for effective communication with healthcare professionals (37).

In light of the effects of age and educational attainment, information support should be tailored according to patients’ risk profiles. For individuals in the low- and moderate-overload groups, intelligent health education systems may provide an effective means of delivering perioperative information. Educational content can be distributed through multiple channels, including official health information platforms, electronic display systems, illustrated educational materials, and artificial intelligence-assisted applications. Organizing information according to the perioperative timeline (preoperative, intraoperative, and postoperative) while presenting content in progressive levels of complexity may improve both accessibility and communication efficiency (39). However, digital approaches alone may be insufficient for patients in the high-information-overload group and, because of the digital divide, could even create additional barriers to information access. For these patients, a human-centered, low-technology approach is likely to be more appropriate. Such strategies may include: (1) involving family caregivers throughout the educational process, using face-to-face counseling supplemented with concise written materials to ensure that essential information is reinforced by caregivers; (2) repeating and reinforcing key educational content through at least two structured review sessions before discharge, thereby avoiding excessive information delivery during a single encounter; (3) providing proactive postoperative support through scheduled telephone follow-up on postoperative days 1 and 7, allowing rehabilitation guidance to be delivered gradually; and (4) coordinating with community healthcare services to provide home-based rehabilitation support for patients with limited access to postoperative care. Together, these high-touch, low-technology interventions complement intelligent educational platforms and create a comprehensive, stratified information support system. Future digital health platforms should also incorporate age-friendly design principles to help reduce disparities in digital access and usability (40).

Differences among surgical specialties were also observed. Patients undergoing gastrointestinal surgery or urologic procedures were more likely to belong to the low-information-overload group than those undergoing orthopedic or hepatobiliary surgery. Gastrointestinal procedures in the present cohort primarily included surgery for thyroid tumors, inguinal hernias, and breast tumors (41–43), whereas urologic procedures mainly involved treatment of urinary calculi (44). These operations are generally minimally invasive, have well-defined indications, are associated with relatively rapid recovery, carry a low risk of postoperative complications, and require comparatively straightforward postoperative care. In contrast, orthopedic procedures (45–47), including surgery for knee ligament injuries, fracture fixation, meniscal injuries, ankle disorders, and lumbar spine disease, encompass a wide range of surgical techniques and are frequently accompanied by substantial postoperative pain, which may interfere with patients’ ability to receive and retain health information. Moreover, orthopedic patients must master extensive rehabilitation instructions, including functional exercise protocols and weight-bearing schedules, while recovery often extends over a prolonged period. Patients undergoing hepatobiliary surgery, many of whom receive interventional treatment for liver cancer, face additional challenges related to the complexity of disease management and prolonged treatment courses. These patients commonly experience embolization-related adverse effects, including abdominal pain, abdominal distension, nausea, and vomiting, and because many are middle-aged or older adults, the burden of symptom management is often considerable (48, 49). Consequently, patients undergoing orthopedic and hepatobiliary procedures may be particularly vulnerable to information overload because of sustained disease management demands and continuous exposure to complex medical information. We therefore recommend that day surgery teams develop specialty-specific educational strategies. For orthopedic and hepatobiliary patients, information should be delivered incrementally throughout the perioperative period, supported by multimodal educational resources integrated within intelligent day surgery platforms. In addition, patients and their families should be encouraged to participate actively in shared decision-making. Tailoring information management to the specific requirements of each surgical specialty may reduce information burden while promoting more effective postoperative recovery.

### Limitations

Several limitations should be acknowledged. First, this investigation was a single-center, cross-sectional study that used convenience sampling, and all participants were recruited from a single institution. Accordingly, the generalizability of the findings to other healthcare settings or patient populations may be limited. In addition, the principal study variables were measured using self-administered questionnaires. Although all instruments demonstrated acceptable reliability and validity, recall bias and social desirability bias cannot be completely excluded. Furthermore, the cross-sectional design precludes conclusions regarding causal relationships. Because LPA is inherently sample-dependent, the stability and reproducibility of the identified latent classes should be confirmed in independent cohorts. Second, the multinomial logistic regression model used the high-information-overload subgroup (*n* = 41) as the reference category. Given the relatively small number of events relative to the number of covariates included in the model, the precision of some parameter estimates may have been reduced, as reflected by the relatively wide confidence intervals observed for several odds ratios. Future investigations should include larger sample sizes and may benefit from applying penalized regression techniques or bootstrap-based internal validation to improve model stability. Furthermore, the distribution of surgical types among the patients enrolled in this study was uneven. Different surgical types may correspond to distinct postoperative recovery experiences, health education needs, and post-discharge support requirements; therefore, the impact of case composition on the study outcomes cannot be entirely ruled out. Therefore, increasing the sample size and conducting a multicenter prospective study are required to validate the robustness and external validity of the current findings.

## Conclusion

Using LPA, this study identified distinct patterns of information overload among patients undergoing day surgery and explored the factors associated with subgroup membership. Three clinically meaningful latent profiles were identified, corresponding to low, moderate, and high levels of information overload. Age, educational attainment, occupation, and surgical specialty were independently associated with profile membership and therefore represent important predictors of patients’ information burden. These findings indicate that perioperative information support should be individualized according to patients’ risk characteristics rather than delivered uniformly to all individuals. Implementing stratified information management strategies designed to reduce unnecessary cognitive burden may improve patients’ ability to process essential health information, facilitate postoperative recovery, and provide an evidence-based framework for precision health education in day surgery practice.

## Data Availability

The original contributions presented in the study are included in the article/supplementary material, further inquiries can be directed to the corresponding author/s.

## References

[1] Bailey CR Ahuja M Bartholomew K Bew S Forbes L Lipp A . Guidelines for day-case surgery 2019: guidelines from the Association of Anaesthetists and the British Association of day Surgery. Anaesthesia. (2019) 74:778–92. doi: 10.1111/anae.14639, 30963557

[2] Sun H Wang K Zhu H Wang Y Lu Y. Considerations of the standardized development of day surgery in China based on the changes of day surgery from 2016 to 2022. Chin Hosp. (2022) 26:10–3.

[3] Lillie H Katz RA Carcioppolo N Giorgi EA Jensen JD. Cancer information overload across time: evidence from two longitudinal studies. Health Commun. (2023) 38:1878–86. doi: 10.1080/10410236.2022.2038866, 35172651 PMC9378766

[4] Eraslan P Ilhan A. Cancer information overload and death anxiety predict health anxiety. Eur Rev Med Pharmacol Sci. (2023) 27:291–8. 36647879 10.26355/eurrev_202301_30902

[5] Arnold M Goldschmitt M Rigotti T. Dealing with information overload: a comprehensive review. Front Psychol. (2023) 14:28. doi: 10.3389/fpsyg.2023.1122200, 37416535 PMC10322198

[6] Sereku P Gencer H Zkan S. Finding useful cancer information may reduce cancer information overload for internet users. Health Inform Librar J. (2020) 37:319–28. doi: 10.1111/hir.12325, 32770732

[7] Eraslan P Tufan G. Cancer information overload may be a crucial determinant of adjuvant aromatase inhibitor adherence. Eur Rev Med Pharmacol Sci. (2022) 26:7053–62. doi: 10.26355/eurrev_202210_29889, 36263553

[8] Wu Q Gao S Dong F Shang J Wang L. Cross-cultural adaptation and validation of the Chinese version of atrial fibrillation information overload scale. Asian Nurs Res. (2025) 19:397–402. doi: 10.1016/j.anr.2025.06.001, 40517952

[9] Thoen CW Sæle M Strandberg RB Eide PH Kinn LG. Patients’ perspectives on postoperative follow-up calls in day surgery: a qualitative study. Scand J Caring. (2025) 39:e70077. doi: 10.1111/scs.70077, 40589287 PMC12209737

[10] Rajala M Kääriäinen M Tanhua A Kaakinen P. Patients’ Perspectives on Postoperative Follow‐Up Calls in Day Surgery: A Qualitative Study. Scandinavian Caring Sciences. (2025) 39:e70077. doi: 10.1111/scs.70077 PMC12209737 40589287

[11] Lim K Smucny J Barch DM Lam M Keefe RSE Lee J. Cognitive subtyping in schizophrenia: a latent profile analysis. Schizophr Bull. (2021) 47:712–21. doi: 10.1093/schbul/sbaa157, 33098300 PMC8084446

[12] Kui Y Jian P Jun Z. The application of latent profile analysis in organizational behavior research. Adv Psychol Sci. (2020) 28:1056–70. doi: 10.3724/SP.J.1042.2020.01056, 40598015

[13] Ni P Chen J Liu N. The sample size estimation in quantitative nursing research. Chin J Nurs. (2010) 45:378–80.

[14] Jensen JD Carcioppolo N King AJ Scherr CL Jones CL Niederdieppe J. The Cancer information overload (CIO)scale: establishing predictive and discriminant validity. Patient Educ Couns. (2014) 94:90–6. doi: 10.1016/j.pec.2013.09.016, 24268921

[15] Ding Y Han J Yang F. Analysis of status quo and influencing factors of information overloadin patients with atrial fibrillation. Chin Nurs Res. (2023) 37:355–8.

[16] Zhang W Zhao YN Liu Y Hao HM Zhang Y Han Y. The mediating effect of information self-efficacy between family care and e-health literacy of community residents. Military Nursing. (2022) 39:29–32.

[17] Guo S Yu X Sun Y Nie D Li X Wang L. Adaptation and evaluation of Chinese version of eHEALS and its usageamong senior high school students. Zhongguo Jian Kang Jiao Yu. (2013) 29:106–108+123.

[18] Zhang Z Wang Y Jian R Wu Z. Latent profiles and influencing factors of perioperative informationoverload in cervical cancer patients. Military Nurs. (2025) 42:83–86+90.

[19] Jiang L Xie X Dai Y Ma H. Pre-administration management standards for day surgery in West China Hospital of Sichuan University. West China Med J. (2019) 34:133–6. doi: 10.7507/1002-0179.201901130

[20] Jiang L Ma H. Core principles of quality and safety management for day surgery at West China Hospital of Sichuan University. West China Med J. (2025) 40:173–7.

[21] Chen Q Li Z Zhao M Huang X. Technology anxiety in elderly patients with chronic co-morbidities: a latent profile analysis. BMC Public Health. (2025) 25:2430. doi: 10.1186/s12889-025-23616-0, 40640790 PMC12243416

[22] Yuen E Winter N Savira F Huggins CE Nguyen L Cooper P . Digital health literacy and its association with sociodemographic characteristics, health resource use, and health outcomes: rapid review. Interact J Med Res. (2024) 13:e46888. doi: 10.2196/46888, 39059006 PMC11316163

[23] An J Wan K Xiang Z Han P Zhu W Li C. From information overload to health information avoidance among Chinese adults aged 50 years and above: the association of self-perceptions of aging and techno-exhaustion. BMC Psychol. (2026). doi: 10.1186/s40359-026-05090-4, 42402598 PMC13613810

[24] Hu H Jin G Zhang X. Older adults and online health misinformation: a systematic literature review. BMC Psychol. (2026) 14:954. doi: 10.1186/s40359-026-04714-z, 42106840 PMC13326225

[25] Kim SH Lim A Benjasirisan C Himmelfarb CR Davidson PM Wright R . Relationships among symptom burden, self-care, and quality of life among individuals living with heart failure and multimorbidity: a cross-sectional study. J Clin Nurs. (2026) 35:3098–112. doi: 10.1111/jocn.70266, 41741378

[26] Benzi IMA La TA Zarbo C Compare ADNS Lazzari D Lingiardi V . Beyond symptoms: a person-centered approach to psychological assessment using transdiagnostic factors. Insights from the PSYCARE study on Italy's 'psychological bonus' policy. J Affect Disord. (2025) 385:119390:40350092. doi: 10.1016/j.jad.2025.119390 40350092

[27] Baxter KA Sachdeva N Baker S. The application of cognitive load theory to the Design of Health and Behavior Change Programs: principles and recommendations. Health Educ Behav. (2025) 52:469–77. doi: 10.1177/10901981251327185, 40145544 PMC12246501

[28] Moschofidou M Racine G Giordani I Chartoumpekis DV Ameti A Sykiotis GP. Comprehensive evaluation of Medtronic's butterfly platform, a new audiovisual information material for patient education and shared decision-making in surgical thyroid disease. BMC Med Inform Decis Mak. (2026) 26:219. doi: 10.1186/s12911-026-03526-w, 42057023 PMC13270795

[29] Liu J Chen X Zhou Y Zhang J Zhang Z Zhang H . Providing older adults experiencing benign prostatic hyperplasia with an education booklet to improve health information recall. J Multidiscip Healthc. (2025) 18:7061–71. doi: 10.2147/JMDH.S552838, 41200080 PMC12587855

[30] Jambor HK Ketges J Otto AL von Bonin M Trautmann-Grill K Teipel R . Communicating cancer treatment with pictogram-based timeline visualizations. J Am Med Inform Assoc. (2025) 32:480–91. doi: 10.1093/jamia/ocae319, 39820364 PMC11833489

[31] Gonella S Delfino C Rolfo M Rizzo A Esposito V Berchialla P . Effects of a video-based preoperative educational intervention plus nurse-led reinforcement discussion on knowledge, self-efficacy, and resilience in patients undergoing major surgery. Clin Nurs Res. (2021) 30:753–61. doi: 10.1177/1054773820986916, 33403866

[32] Assaf N Bazzi H Ghanem K Saasouh W. Optimizing surgical outcomes through increased familial support in the perioperative period. Front Anesthesiol. (2025) 4. doi: 10.3389/fanes.2025.1650491

[33] Gullet A Tastan S. The effect of discharge training based on teach-Back method on discharge readiness and satisfaction: a randomized controlled trial. Worldviews Evid-Based Nurs. (2025) 22:e70062. doi: 10.1111/wvn.70062, 40698847 PMC12285212

[34] Zanforlini BM Sambo S Devita M Cignarella A Vezzali F Sturani S . A multidisciplinary approach to improve adherence to medical recommendations in older adults at hospital discharge: the APPROACH study protocol. PLoS One. (2024) 19:e0297238. doi: 10.1371/journal.pone.0297238, 38687693 PMC11060519

[35] Markovich L Sela Y Grinberg K. Continuity of care across hospital-to-community transitions: a narrative review integrating concepts, measurement, and nursing-relevant approaches. Healthcare (Basel). (2026) 14:656. doi: 10.3390/healthcare14050656, 41827610 PMC12985294

[36] Wang S Wang Y Niu W Yang H. The current status and influencing factors of the coexistence of multiple frailty domains in patients with chronic heart failure. Chin J Nurs. (2025) 60:311–8.

[37] Li Y Li L Wang L. Analysis on Chinese residents' health literacy level: 2012-2023. Chin J Health Educ. (2025) 41:426–30.

[38] Fang Z Zhao Z Cao X. Perceived cognitive slowing and online health information seeking among older adults: motivational beliefs as pathways and community IT culture as a buffer. Front Public Health. (2026) 14:1875038. doi: 10.3389/fpubh.2026.1875038, 42433400 PMC13349898

[39] Huang C Pan H Zhang Z Zhuang Y. Nursing experience in the construction of smart hospital. Chin Nurs Manag. (2025) 25:167–70.

[40] Shi Z Du X Li J Hou R Sun J Marohabutr T. Factors influencing digital health literacy among older adults: a scoping review. Front Public Health. (2024) 12:1447747. doi: 10.3389/fpubh.2024.1447747, 39555039 PMC11566617

[41] Li X Zheng X Jiang K. Chinese expert consensus on thyroid day surgery (2021 edition). China J Gen Surg. (2021) 30:499–509.

[42] Jiang F Liu Y Wang M. Precautions for patients with comorbidities undergoing inguinal hernia day surgery. Chin J Pract Surg. (2023) 43:662–6.

[43] Ling R Ma L Liu Y Liu Y Zhang J. Chinese expert consensus on the ambulatory surgery of breast surgery (2021 edition). Chin J Pract Surg. (2021) 41:1201–5.

[44] Xuan H Chen Q Zhong H Cao Y Xia L Xue W. Feasibility analysis of daytime surgical mode of holmium laser lithotripsy under ureteroscopy. Chin J Laser Med Surg. (2018) 27:115.

[45] Xie J Yang J Huang Q Li J Li T Diao Y . Expert consensus on the management of perioperative pain, sleep, and anxiety disorders in orthopaedics and anesthesiology for enhanced recovery after surgery. Chin J Bone Joint Surg. (2025) 18:212–21.

[46] Cui B Gu H Zhang X. Application of wearable devices in home rehabilitation care for patientsafter anterior cruciate ligament reconstruction surgery. J Nurs Sci. (2024) 39:93–6.

[47] Wen Y Guo X Sun G Liu Q Qin D. Application effect of orthopedic specialist nurse-led "internet+ medicahealth" intervention in patients with anterior cruciate ligament reconstruction. Chin Nurs Res. (2023) 37:2043–8.

[48] Li D Yang L Qiu L Tan Z Wu L Chen X. Analysis of the factors influencing the status of coexistence with cancerin young and middle-aged HCC patients after receiving interventional therapy. J Interv Radiol. (2025) 34:772–6.

[49] Pan Y Chang R He Z Hong M. How to prophylactically alleviate postembolization syndrome following transarterial chemoembolization: protocol of a double blinded, randomized, placebo-controlled trial. Medicine (Baltimore). (2021) 100:e25360. doi: 10.1097/MD.0000000000025360, 33832116 PMC10545327

